# High SARS-CoV-2 Prevalence among Healthcare Workers in Cochabamba, Bolivia

**DOI:** 10.3390/v14020232

**Published:** 2022-01-25

**Authors:** Paola Mariela Saba Villarroel, María del Rosario Castro Soto, Verónica Undurraga, Heydi Sanz, Ana María Jaldín, Laetitia Ninove, Elif Nurtop, Laura Pezzi, Souand Mohamed Ali, Abdennour Amroun, Morgan Seston, Xavier de Lamballerie

**Affiliations:** 1 Aix-Marseille Univ, IRD 190, Inserm 1207, Unité des Virus Émergents (UVE), 27 Boulevard Jean Moulin, 13005 Marseille, France; Laetitia.NINOVE@ap-hm.fr (L.N.); enurtop@gmail.com (E.N.); laura.pezzi3@studio.unibo.it (L.P.); souand.mohamedali@gmail.com (S.M.A.); abdennour.amroun@inserm.fr (A.A.); morgan.seston@ird.fr (M.S.); xavier.de-lamballerie@univ-amu.fr (X.d.L.); 2 Infectology Department, Viedma Hospital, Cochabamba 4780, Bolivia; rossycastrosoto@hotmail.com; 3 María de los Ángeles Clinic, Cochabamba 4780, Bolivia; draveronicaundurraga@aol.com; 4 Manuel Ascencio Villarroel Hospital, Cochabamba 4780, Bolivia; heydi.sanz@gmail.com; 5 Clínica Copacabana, Cochabamba 4780, Bolivia; anamajaldin@hotmail.com

**Keywords:** seroprevalence, SARS-CoV-2, healthcare workers, Bolivia

## Abstract

Healthcare workers (HCWs) are at increased risk of SARS-CoV-2 infection. The aim of the study was to estimate the SARS-CoV-2 seroprevalence among HCWs in Cochabamba, Bolivia and to determine the potential risk factors. In January 2021, a cross-sectional SARS-CoV-2 seroprevalence study was conducted in 783 volunteer clinical and non-clinical HCWs in tertiary care facilities. It was based on IgG detection using ELISA, chemiluminiscence, and seroneutralisation tests from dried blood spots. Analysis revealed a high seroprevalence (43.4%) of SARS-CoV-2 IgG antibodies. The combination of anosmia and ageusia (OR: 68.11; 95%-CI 24.83–186.80) was predictive of seropositivity. Belonging to the cleaning staff (OR: 1.94; 95%-CI 1.09–3.45), having more than two children in the same house (OR: 1.74; 95%-CI 1.12–2.71), and having been in contact with a close relative with COVID-19 (OR: 3.53; 95%-CI 2.24–5.58) were identified as risk factors for seropositivity in a multivariate analysis. A total of 47.5% of participants had received medication for COVID-19 treatment or prevention, and only ~50% of symptomatic subjects accessed PCR or antigenic testing. This study confirms a massive SARS-CoV-2 attack rate among HCWs in Cochabamba by the end of January 2021. The main risk factors identified are having a low-skilled job, living with children, and having been in contact with an infected relative in the household.

## 1. Introduction

Since December 2019, the COVID-19 pandemic has represented a major crisis worldwide. Particularly in developing countries, the health system is on the verge of collapse, and the shortage of oxygen supply and healthcare workers (HCWs) [1] makes management particularly challenging. In addition, transmission is exacerbated by the shortage of personal protective equipment and diagnostic tests.

In Bolivia, the first confirmed case of coronavirus disease 2019 (COVID-19) was reported on 10 March 2020 [2], followed by a nationwide lockdown that began on 22 March and ended on 10 May [3]. In total, national authorities reported 160,124 confirmed positive cases, with 9165 deaths by the end of 2020 [4]. To date, three epidemic waves have been reported in Bolivia: the first epidemic peak occurred in July and August 2020, the second peak occurred in January and early February 2021, and a third peak occurred in May and June 2021 [5].Vaccination campaigns for COVID-19 began on 29 January 2021, with doses reserved for HCWs [6].

Cochabamba is the fourth largest city in Bolivia, with ~826,316 inhabitants in 2020 [7]. It is located in a valley in the Andes mountain range. Cochabamba has been one of the most affected cities in Bolivia during the COVID-19 pandemic, with frequent cases among HCWs in June 2020, leading to the closure of some hospitals.

In this context, we conducted a seroprevalence study among HCWs in Cochabamba, Bolivia to estimate exposure and the risk factors associated with SARS-CoV-2 infection.

## 2. Materials and Methods

### 2.1. Study Design and Participants

In January 2021, during the second epidemic wave and before the start of vaccination campaigns, 783 clinical and non-clinical HCWs from different healthcare facilities in Cochabamba, Bolivia agreed to participate in this cross-sectional study.

Participants were included from: (i) the main and largest public tertiary-care hospitals admitting COVID-19 patients: Viedma adult hospital (360 of 826 employees; participation rate 43.6%) and Manuel Ascencio Villarroel children’s hospital (206/410; 50.2%); (ii) two private tertiary care clinics: Copacabana Clinic (81/97; 83.5%) and María de los Ángeles Clinic (71/75; 94.7%), both of which also admit COVID-19 patients; (iii) sixty-five additional HCWs were included from Central Diagnostic Laboratory, Germán Urquidi Maternity Hospital, and Cochabamba Clinic.

All participants were included, irrespective of whether they had had COVID-19 symptoms or not. All consenting subjects were asked to answer a questionnaire and to provide a fingerstick capillary blood sample on blood cards.

The questionnaire allowed for collection of information concerning demographics, occupation, hospital department, working time since the beginning of the pandemic (including lockdown), household size and composition, blood type, tobacco smoking, types of face mask used (surgical, FFP2/KN95, cloth), contacts with confirmed COVID-19 cases (patients, family, friends, and/or colleagues), symptoms since the beginning of the pandemic (ageusia, anosmia, or at least two of the followings: fever, cough, tiredness, rhinitis, sore throat, headache, conjunctivitis, or diarrhea), comorbidities, previous COVID-19 diagnosis (PCR, antigen, and serology tests), hospitalization, treatment, and chlorine-dioxide ingestion.

### 2.2. Ethical Approval

The ethics committee of the Dr. Mario Ortíz Suárez hospital in Bolivia (No. 09/2020) approved this study. HCWs agreed to participate in the study by providing written informed consent prior to enrollment. Blood samples, personal data, and biological results were irreversibly anonymized.

### 2.3. Serological Testing

Sera were recovered from dried blood spots (DBS) using a standardized quantitative elution protocol. Briefly, four 4.7 mm discs were punched and stored in 0.5 mL 2D FluidX 96-format tubes (Brooks life sciences, Chelmsford, MA, USA) [8]. Elution was performed with 380 µL of PBS, shaken at 1050 rpm for one hour and left for 18 h at room temperature before analysis. The sample eluates were tested with three different SARS-CoV-2 serological tests: (i) a semi-quantitative chemiluminescent immunoassay (CLIA) detecting IgG against the receptor binding domain (RBD) (Access SARS-CoV-2 IgG II, Beckman, CA, USA), run on the DXI instrument; (ii) a commercial semi-quantitative ELISA detecting IgG against the S1 domain of the spike protein (Anti-SARS-CoV-2, EUROIMMUN, Lübeck, Germany; sensitivity 87%, specificity 97.5% [8]), run on the EUROLabWorkstation instrument; (iii) a highly specific in-house virus-neutralization test (VNT) cytopathic effect (CPE)-based, 100 TCID50 of the SARS-CoV-2 BavPat1 strain, with serum dilutions from 1:20 to 1:160, as previously described [9]. The two commercial assays were performed according to the manufacturer’s instruction. However, for the EUROIMMUN S1 ELISA assay, the cut-off ratio to define equivocal results was decreased to 0.7 (instead of 0.8). CPE-based VNT was performed for all samples with either a positive or equivocal ELISA result or a positive CLIA result.

### 2.4. Diagnostic Interpretation

Samples were considered positive when at least two tests were positive among the S1 ELISA (Ratio ≥ 1.1), the CLIA RBD assay (titre > 10 AU/mL), and the VNT test (titre ≥ 40).

### 2.5. Statistical Analysis

For analysis, participants were assigned to four age groups (18–30 years old (yo); 31–40 yo; 41–60 yo; >60 yo) and to clinical or non-clinical occupational groups. Clinical HCWs (532/780, 68.2% of the study population) included physicians (*n* = 96, 12.3%), nurses (*n* = 91, 11.7%), assisting nurses (*n* = 83, 10.6%), laboratory staff (*n* = 82, 10.5%), medical students (*n* = 80, 10.3%), resident physicians (*n* = 56, 7.2%), physiotherapists and kinesiologists (*n* = 20, 2.6%), radiologists (*n* = 12, 1.5%), and nutritionists (*n* = 12, 1.5%). Non-clinical HCWs (248/780, 31.8%) included administrative staff (*n* = 109, 14.0%), cleaning (*n* = 88, 11.3%), kitchen (*n* = 11, 1.4%), and maintenance (*n* = 10, 1.3%) staff, security guards (*n* = 10, 1.3%), psychologists (*n* = 6, 0.8%), and others (*n* = 14, 1.8%). For test accuracy, equivocal ELISA results were excluded from analysis and were analyzed according to our stablished criteria for seropositivity. Cohen’s Kappa test was used to calculate the degree of accuracy between commercial assays.

Continuous variables are presented as means with standard deviations (SD), and categorical variables as numbers and percentages with odds ratio (OR) and 95% confidence interval (CI) when relevant. Variables were compared using the Pearson’s chi-square test to identify those associated with the presence or absence of IgG antibodies against SARS-CoV-2. Variables used to evaluate risk factors for seropositivity were age, sex, hospital, occupation, clinical or non-clinical occupational group, working during lockdown, household and composition, blood type, tobacco smoking, face-mask types used, and COVID-19 contact. Variables with *p*-values < 0.05 were considered statistically significant (Bonferroni-adjusted *p* < 0.05) and were included in multivariate logistic-regression analysis to determine whether each variable was an independent factor for seropositivity. Analyses were performed using IBM-SPSS Statistics v 24.0.0.0 (Chicago, IL, USA) and GraphPad Prism 7.00 (San Diego, CA, USA) software.

## 3. Results

### 3.1. Characteristics of the Study Population

The mean age of participants was 39.29 (SD 12.3) years, and participants were predominantly female (579/783, 73.9%). The O blood type was the most common, followed by the A blood type (76.7% and 16.7%, respectively, in 708 participants, who provided the information). An underlying disease was reported by 23.1% of participants (including diabetes 32/783, 4.1%; hypothyroidism 30/783, 3.83%; hypertension 27/783, 3.4%; and Chagas disease 23/783, 2.9%). Sixty-seven of 760 (8.8%) participants were tobacco smokers.

The mean household size was 3.1 (SD 1.64), with the following composition: children < 11 years, mean 2.74 (SD 1.39); persons ≥ 11 years old, 3.42 (SD 1.75). The mean number of bedrooms was 3.97 (SD 1.65).

A proportion of HCWs wore surgical masks exclusively (311/783, 39.7%), FFP2/KN95 masks exclusively (103/783, 13.2%), or a combination of surgical and FFP2/KN95 masks (303/783, 38.7%). Among clinical HCWs, 412/532 (77.4%) reported contact with COVID-19 patients, in contrast to 115/248 (46.4%) among non-clinical HCWs, with higher proportions in nurses and assisting nurses (~83%), as well as physicians (82%). Household contact with a COVID-19 case was declared by 158/783 (20.2%) participants.

Most of the study population (656/769, 85.3%) worked during the lockdown, and 24/763 (3.1%) were hospitalized due to COVID-19. Details of demographics, occupational exposure, symptoms, and tobacco smoking are presented in Table 1, Tables S1 and S2. Of note, prior to sample collection, among the health care facilities involved in the study, ten deaths were reported, and six HCWs did not return to work permanently due to COVID-19 sequelae.

### 3.2. SARS-CoV-2 Seroprevalence

Overall, 340/783 (43.4%; 95% CI 38.8–48.0) participants were seropositive for SARS-CoV-2 IgG antibodies. There was no significant difference in sex, blood type, healthcare facility, or type of protective masks used.

**Figure 1:**
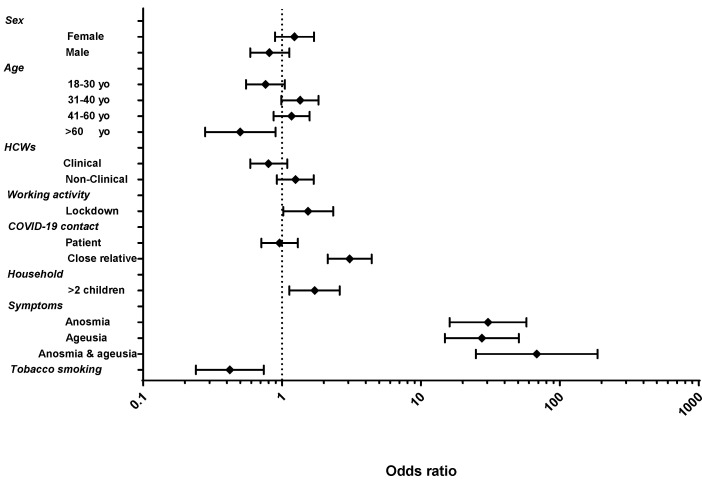
Odds ratio (CI 95%) of SARS-CoV-2 seropositivity in healthcare workers. Cochabamba (Bolivia), January 2021.

**Figure 2:**
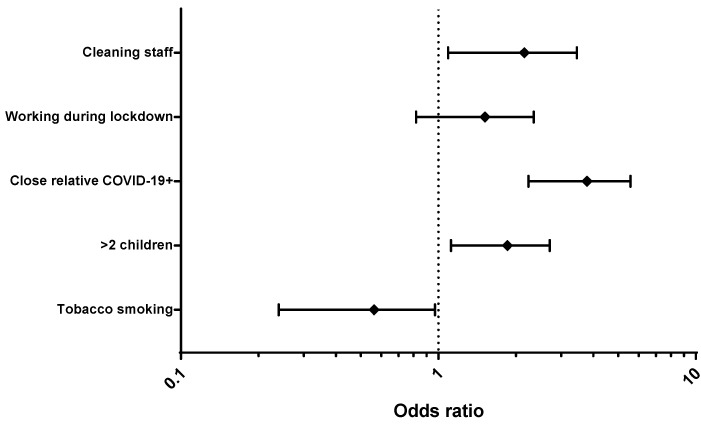
Multivariate analysis of SARS-CoV-2 seroprevalence (significant *p*-values) -associated factors in healthcare workers. Cochabamba, Bolivia. January 2021. Data are presented as odds ratio with CI 95%.

In univariate model, according to occupation, cleaning workers were associated to a higher seroprevalence (58%, OR: 1.94; 95% CI 1.24–3.03). Seropositivity was associated with having more than two children living in the same house (53.4%, OR: 1.71; 95% CI 1.13–2.60), having been in contact with a close relative with COVID-19 (65.2%, OR: 3.07; 95% CI 2.13–4.42), and working during lockdown (44.8%, OR: 1.54; 95% CI 1.02–2.34). Of note, tobacco smoking was negatively associated with seropositivity (25.4%, OR: 0.420; 95% CI 0.24–0.74) (Figure 1). According to multivariate analysis, belonging to the cleaning staff, having more than two children living in the same house, and being in contact with a close relative with COVID-19 were significantly associated with SARS-CoV-2 seropositivity, and smoking was significantly associated with seronegativity (Table S3 and Figure 2).

Among seropositive participants, 22.9% (76/332) did not report any of the symptoms mentioned in the questionnaire. Anosmia (OR: 30.42; 95% CI 16.09–57.50) and the combination of anosmia and ageusia (OR: 68.11; 95% CI 24.83–186.80) were highly predictive of SARS-CoV-2 seropositivity (Figure 1). Of participants who reported symptoms or were seropositive for IgG, ~50% had a history of previous PCR and/or antigen testing. Participants who reported an underlying disease were 4.14 times more likely to be hospitalized (OR: 4.14; 95% CI 1.82–9.42).

### 3.3. Diagnostic Tests

Three hundred nineteen out of 340 (93.8%) seropositive participants had positive results for all three assays (ELISA, CLIA, and VNT). For ELISA and CLIA tests, the sensitivity was 98.2% and 97.0%, the specificity was 97.3% and 99.0%, the positive predictive value was 97.0% and 98.8%, and the negative predictive value was 98.4% and 96.5%, respectively, with almost perfect agreement between both tests (Cohen’s k: 0.91). In VNT, only positive or equivocal ELISA and CLIA results were tested, showing a VNT sensitivity and specificity of 100%.

### 3.4. Treatment

A total of 47.5% (315/663) of HCWs had received at least one type of medication, mainly ivermectin (224/663, 33.8%), azithromycin (179/663, 27.0%), and corticosteroids (53/663, 7.9%), and more frequently in symptomatic (225/350, 64.3%) than in asymptomatic participants (85/296, 28.7%).

Oral chlorine dioxide was used by 83/735 (11.3%) HCWs, more often in non-clinical HCWs (39/222, 17.6%) than clinical HCWs (44/510, 8.6%), and more often in symptomatic (53/386, 13.7%) than asymptomatic participants (29/327, 8.7%).

## 4. Discussion

We found that the SARS-CoV-2 attack rate was massive among clinical and non-clinical HCWs from Cochabamba, Bolivia by the end of January 2021, with a seroprevalence of 43.4% documented by a combination of IgG detection tests with high sensitivity and specificity. This is, as far as we know, the highest rate reported from seroprevalence studies performed among HCWs in Latin America in 2020: 0.75–0.9% in Argentina [10,11], 5.5–21.4% in Brazil [12,13,14,15], 2.0–24% in Chile [16,17], 8.3% [18] in Colombia, 6–20% in Mexico [19,20], 11.6% in Panama [21]. A single study reported a very high seroprevalence (58.3%) in Peru [22], but it relied on rapid tests.

Consistent with previous reports in the international literature, anosmia and the combination of anosmia and ageusia were the most specific predictive symptoms associated with evidence for previous SARS-CoV-2 seropositivity [23,24]. A total of 22.9% of seropositive participants did not mention any symptoms [23,25]. The number of asymptomatic cases and the lack of diagnosis in almost 50% of symptomatic HCWs could have contributed to a substantial number of secondary infections and to the high seroprevalence rates observed.

In univariate analysis, seropositivity did not differ by sex, health facility, age, or the type of protective mask used. In multivariate analysis, whilst clinical HCWs were more exposed to COVID-19 patients than non-clinical HCWs (77.4% vs. 46.4%), cleaning workers were associated with the highest seroprevalence (57.9%), as previously described [26,27]. This excess risk in low-income occupational groups may be related to their frequent use of public transportation, less adherence to social distancing and the use of face masks, living in overcrowded housing (50% lived with more than five persons in our study), and limited access to diagnostic tests. Being in contact with a close relative with COVID-19 and living with more than two children were also significantly associated with seropositivity. As previously reported [8], children may play an important role in household transmission, reinforced by the frequent occurrence of asymptomatic infections [28].

Active tobacco smokers exhibited lower IgG seroprevalence compared with non-smokers [8,29,30,31]. This should not be taken as a false message to promote smoking to avoid infection [32]. On the one hand, tobacco smoking is independently associated with a clear excess risk of severe disease (e.g., cancer, respiratory and cardiovascular diseases), and, on the other hand, it is possible that tobacco smokers infected with SARS-CoV-2 are prone to develop severe disease [33]. Previous studies identified a lower risk of SARS-CoV-2 infection among people with blood type O [9,34,35,36,37]. We did not find this trend in our study, but the overwhelming proportion of blood group O in Bolivia [38] (76.7% in our study) makes this type of assessment difficult and suggests that larger studies are needed to precisely assess this association.

In Bolivia, the spread of COVID-19 was accompanied by great concern among the general population, misinformation, and common self-medication [39]. The antiparasitic ivermectin and the antibiotic azithromycin, alone or combined with the anti-malarial hydroxychloroquine, grabbed attention [40,41] and were widely used as treatment or prophylaxis for SARS-CoV-2 infection after having shown some antiviral activity in vitro [42,43] and sometimes recommended by the health authorities [44]. We found that ivermectin (33.8%) and azithromycin (26.9%) were frequent used (also occurred among HCWs) amid the fear of contracting the disease (28.7% in asymptomatic participants) or to treat it (64.3% in symptomatic participants). In addition, chlorine dioxide, a derivate of bleach, which is potentially toxic and can cause serious and potentially life-threating side effects, falsely marketed as “Miracle Mineral Solution” for prevention and treatment of COVID-19 [45], has been commonly used by the Bolivian population (11.3% in our study) and even promoted by health authorities [1].

Our study has some limitations. It was carried out among volunteer healthcare workers, which may have resulted in a bias towards those who have already been infected with SARS-CoV-2, either presumed or confirmed. Therefore, the prevalence figures provided should be considered as order-of-magnitude indications. On the other hand, the strength of this research program lies in the consistency of the information collected (with reference to other international studies and to what is known about the dynamics of the epidemic in Bolivia and the behavior of the population) and in the possibility of collecting seroprevalence data prior to the implementation of vaccination campaigns.

## 5. Conclusions

In conclusion, this study shows high seroprevalence rates among HCWs in Cochabamba, Bolivia, suggesting that the first wave of COVID-19 had a considerable impact on this occupational group. Better information and protective measures, continuous awareness and sensibilization, and a greater access to diagnostic tests are needed to reduce the risk of infection.

## Figures and Tables

**Table 1:** SARS-CoV-2 seropositivity by demographics, occupational exposure, symptoms, and tobacco smoking in healthcare workers. Cochabamba (Bolivia), January 2021.

Variables		Total	Seropositive	*p*-Value
Age				
	Age (years), mean (SD)	39.29 (12.3)	39.18 (11.5)	
	18–30, *n* (%)	220	85 (38.64)	0.09
	31–40, *n* (%)	241	117 (48.55)	0.05
	41–60, *n* (%)	263	121 (46.01)	0.30
	>60, *n* (%)	59	17 (28.81)	0.02
Sex				
	Female, *n* (%)	579	259 (44.73)	0.21
	Male, *n* (%)	204	81 (39.71)	
Healthcare facility				
	Viedma Adult Hospital, *n* (%)	360	150 (41.67)	0.36
	Manuel Ascencio Villarroel Hospital, *n* (%)	206	93 (45.15)	0.32
	Copacabana Clinic, *n* (%)	81	31 (38.27)	0.56
	María de los Ángeles Clinic, *n* (%)	71	36 (50.70)	0.19
	Others, *n* (%)	65	30 (46.15)	0.65
Occupation				
	Clinical HCWs, *n* (%)	532	222 (41.73)	0.15
	Non-clinical HCWs, *n* (%)	248	117 (47.20)	
	Missing, *n* (%)	3	1	
Household composition				
	>two children, *n* (%)	116	62 (53.45)	**0.01**
	≤two children, *n* (%)	407	163 (40.05)	
	Missing, *n* (%)	260	115	
Tobacco smoking				
	Active tobacco smokers, *n* (%)	67	17 (25.37)	**0.02**
	No smoking, *n* (%)	693	310 (44.73)	
	Missing, *n* (%)	23	13	
COVID-19 contact				
	Patient, *n* (%)	529	228 (43.10)	NA
	Relative living in the same house, *n* (%)	158	103 (65.19)	
	Relative not living in the same house, *n* (%)	109	37 (33.94)	
Symptoms				
	No symptoms, *n* (%)	348	76 (21.84)	**<0.001**
	Symptoms ^1^, *n* (%)	406	256 (63.05)	
	Missing, *n* (%)	29	8	
Working activity				
	Working during lockdown, *n* (%)	656	294 (44.82)	**0.041**
	Not working during lockdown, *n* (%)	113	39 (34.51)	
	Missing, *n* (%)	14	8	

^1^ Ageusia, anosmia, or at least two of the followings: fever, cough, tiredness, rhinitis, sore throat, headache, conjunctivitis, or diarrhea. Abbreviations: *n*: number; NA: Not applicable; SD: Standard Deviation. Significant values are in bold (after Bonferroni correction for variables for more than 2 categories).

## Data Availability

The data that support the findings of the study are available within the article. Additional data are available upon request from the corresponding author.

## Supplementary Materials

The following are available online at https://www.mdpi.com/article/10.3390/v14020232/s1, Table S1: Demographics, occupational exposure, symptoms, and tobacco smoking in healthcare workers according to SARS-CoV-2 seroprevalence. Cochabamba (Bolivia), January 2021. Table S2: Diagnostic tests performed and medication used before sample collection by healthcare workers. Cochabamba (Bolivia), January 2021. Table S3: Multivariate analysis of SARS-CoV-2 seroprevalence-associated factors in healthcare workers (including significant variables). Cochabamba (Bolivia), January 2021.
