# Supplementary material for: High SARS-CoV-2 Prevalence among Healthcare Workers in Cochabamba, Bolivia
Source: Viruses. 2022 Jan 25;14(2):232. doi: 10.3390/v14020232 (PMC8874891; doi:10.3390/v14020232)
Supplement: Supplementary file 1 [file viruses-14-00232-s001.zip › viruses-1514082-supplementary.pdf]

Supplementary information

High SARS-CoV-2 prevalence among healthcare workers in Cochabamba, Bolivia

Table S1: Demographics, occupational exposure, symptoms, and tobacco smoking in healthcare workers according to SARS-CoV-2 seroprevalence. Cochabamba (Bolivia), January 2021.

Variables		All n=783 *n* (%)	Seropositive n=340 *n* (%)	Seronegative n=443 *n* (%)	p-value
Age (years)					
	18-30	220 (28.1)	85 (25.00)	135 (30.47)	0.09
	31-40	241 (30.8)	117 (34.41)	124 (27.99)	0.05
	41-60	263 (33.6)	121 (35.59)	142 (21.05)	0.30
	>60	59 (7.5)	17 (5.00)	42 (9.48)	0.02
Sex					
	Female	579 (73.9)	259 (76.18)	320 (72.23)	0.21
	Male	204 (26.1)	81 (23.82)	123 (27.77)	
Healthcare facility					
	Viedma Adult Hospital	360 (46.0)	150 (44.1)	210 (47.4)	0.36
	Manuel Ascencio Villarroel Hospital	206 (26.3)	93 (27.3)	113 (25.5)	0.32
	Copacabana Clinic	81 (10.3)	31 (9.2)	50 (11.3)	0.56
	María de los Ángeles Clinic	71 (9.1)	36 (10.6)	35 (7.9)	0.19
	Others	65 (8.3)	30 (8.8)	35 (7.9)	0.65
Healthcare workers					
	Clinical	532/780 (68.2)	222/339 (65.49)	310/441 (70.29)	0.18
	Non-clinical	248/780 (31.8)	117/339 (34.51)	131/441 (29.71)	
Occupation					
	Physicians	96 (12.26)	32 (9.41)	64 (14.45)	0.033
	Nurses	91 (11.62)	46 (13.53)	45 (10.16)	0.145
	Assisting nurses	83 (10.60)	43 (12.65)	40 (9.03)	0.103
	Laboratory staff	82 (10.47)	34 (10.00)	48 (10.84)	0.705
	Medical students	80 (10.22)	23 (6.76)	57 (12.87)	0.005
	Resident physicians	56 (7.15)	24 (7.06)	32 (7.22)	0.929
	Physiotherapists/kinesiologists	20 (2.55)	11 (3.24)	9 (2.03)	0.289
	Radiologists	12 (1.53)	5 (1.47)	7 (1.58)	0.902
	Nutritionists	12 (1.53)	4 (1.18)	8 (1.81)	0.477
	Administrative staff	109 (13.92)	39 (11.47)	70 (15.80)	0.083
	Cleaning staff	88 (11.24)	51 (15.00)	37 (8.35)	**0.003**
	Kitchen staff	11 (2.40)	8 (2.35)	3 (0.68)	0.050

	Maintenance staff	10 (1.28)	7 (2.06)	3 (0.68)	0.088
	Security guards	10 (1.28)	4 (1.18)	6 (1.35)	0.826
	Psycologists	6 (0.77)	2 (0.59)	4 (0.90)	0.251
	Others	14 (1.79)	6 (1.76)	8 (1.81)	0.956
	Missing	3 (0.38)	1 (0.29)	2 (0.45)	0.724
COVID-19 contact					
	Patient	529 (67.6)	228 (67.06)	301 (67.95)	0.79
	Relative living in the same house	158 (20.2)	103 (30.29)	55 (12.42)	**<0.001**
Working activity					
	Working during lockdown	656/769	294/333 (88.29)	362/436 (83.03)	**0.04**
Blood type					
	Blood type O	543 (69.35)	250 (73.53)	293 (66.14)	0.027
	Blood type A	118 (15.07)	43 (12.65)	75 (16.93)	0.097
	Blood type B	39 (4.98)	13 (3.82)	26 (5.87)	0.192
	Blood type AB	8 (1.02)	5 (1.47)	3 (0.68)	0.274
	missing	75 (9.58)	29 (8.53)	46 (10.38)	0.382
Mask type					
	FFP2/KN95 mask	103 (13.15)	40 (11.76)	63 (14.22)	0.313
	Surgical mask	311 (39.72)	143 (42.06)	168 (37.92)	0.241
	FFP2/KN95 and surgical mask	303 (38.70)	130 (38.24)	173 (39.05)	0.054
	Others	57 (7.28)	24 (7.06)	33 (7.45)	0.043
	Missing	9 (1.15)	3 (0.88)	6 (1.35)	0.539
Symptoms					
	Anosmia	160/754 (21.2)	149/332 (44.88)	11/422 (2.61)	**<0.001**
	Ageusia	160/754 (21.2)	148/332 (44.58)	12/422 (2.84)	**<0.001**
	Anosmia & ageusia	135/754 (17.9)	131/332 (39.46)	4/422 (0.90)	**<0.001**
Tobacco smoking		67/760 (8.8)	17/327 (5.19)	50/433 (11.55)	0.002
Comorbidities		181 (23.11)	77 (22.65)	104 (23.48)	0.785

Significant values are in bold (after Bonferroni correction for variables for more than 2 categories)

Abbreviations: *n*: number

Table S2: Diagnostic tests performed and medication used before sample collection by healthcare workers. Cochabamba (Bolivia), January 2021.

**Variables**	**All**<br>***n*** **=783**<br>***n*** **(%)**	**Seropositive**<br>***n*** **=340**<br>***n*** **(%)**	**Seronegative**<br>***n*** **=443**<br>***n*** **(%)**
Diagnostic tests (positive or negative)			
Participants with a PCR or Antigen test	324 (41.38)	160 (47.06)	164 (37.02)
PCR	232 (29.63)	121 (35.59)	111 (25.06)
Antigen	141 (18.01)	59 (17.35)	82 (18.51)
Rapid IgM	223 (28.48)	96 (28.24)	127 (28.67)
Rapid IgG	219 (27.97)	94 (27.65)	125 (28.22)
ELISA/CLIA IgM	155 (19.80)	80 (23.53)	75 (16.93)
ELISA/CLIA IgG	151 (19.28)	80 (23.53)	71 (16.03)
Treatment			
Ivermectin	224/663 (33.79)	131/299 (43.81)	93/364 (25.55)
Azithromycin/azithromycin + hydroxychloroquine	179/663 (27.00)	138/299 (46.15)	41/364 (11.26)
Corticosteroid	53/663 (7.99)	49/299 (16.39)	4/364 (1.10)
Hydroxychloroquine	19/663 (2.87)	13/299 (4.35)	6/364 (1.65)
Favipiravir	5/663 (0.75)	5/299 (1.67)	0/364 (0.00)
Convalescent plasma	4/663 (0.60)	4/299 (1.34)	0/364 (0.00)
Remdesivir	1/663 (0.15)	1/299 (0.33)	0/364 (0.00)
Ribavirin	1/633 (0.15)	1/299 (0.33)	0/364 (0.00)
Interferon	1/633 (0.15)	1/299 (0.33)	1/364 (0.27)
Don't remember	20/633 (3.02)	16/299 (5.35)	4/364 (1.10)
No treatment	348/633 (52.49)	104/299 (34.78)	244/364 (67.03)
Chlorine dioxide	83/735 (11.29)	47/320 (14.69)	36/415 (8.67)

*Fever, cough, tiredness, rhinitis, sore throat, headache, conjunctivitis or diarrhea
Abbreviations: n: number

Table S3: Multivariate analysis of SARS-CoV-2 seroprevalence associated factors in healthcare workers (including significant variables). Cochabamba (Bolivia), January 2021

**Variable**	**Odds ratio (95% CI)**	**p-value**
>2 children living in the same house	1.74 (1.12-2.71)	**0.014**
Cleaning staff	1.94 (1.09-3.45)	**0.025**
Working during lockdown	1.38 (0.82-2.35)	0.225
Tobacco smoking	0.48 (0.24-0.97)	**0.042**
COVID-19 contact/relative living in the same house	3.53 (2.24-5.58)	**<0.001**
